# Analysis of Factors Influencing Downtime During Continuous Renal Replacement Therapy in Critically Ill Patients and Construction of a Prediction Model

**DOI:** 10.1111/nicc.70664

**Published:** 2026-08-27

**Authors:** Bingbing Pang, Jie Zhang, Yanshuo Wu, Qiaoju Kang, Kaihua Dong, Suzhi Guo, Yanling Yin

**Affiliations:** ^1^ Department of Intensive Care Medicine The Fourth Hospital of Hebei Medical University Shijiazhuang China

**Keywords:** continuous renal replacement therapy, critical care nursing, downtime, influencing factors, prediction model

## Abstract

**Background:**

Prolonged CRRT downtime compromises treatment efficacy and worsens outcomes. Daily downtime > 20% exacerbates acidosis and is associated with 28‐day mortality.

**Aims:**

This study aimed to develop and validate a bedside‐accessible nomogram for early identification of treatment days at risk of suboptimal CRRT downtime control in critically ill patients.

**Study Design:**

This retrospective cohort study was conducted in one Chinese ICU (January 2019–December 2023). The binary outcome was cumulative downtime > 2.4 h per standardised treatment day. Variables included demographics, laboratory parameters, CRRT circuit and other treatment factors. GEE identified predictors; the model was displayed as a nomogram and assessed by AUC, calibration and decision curve analysis.

**Results:**

A total of 145 patients contributing 595 CRRT treatment days were included. Total treatment time was 14 280 h and cumulative downtime was 1725.07 h. Mean daily downtime was 2.90 ± 2.72 h (12.08% ± 11.35% of treatment time). Suboptimal CRRT downtime control occurred on 232 treatment days (39.0%). Multivariable GEE analysis identified five independent risk factors for suboptimal downtime control: daily number of filter replacements (OR = 2.629, 95% CI 1.822–3.794, *p* < 0.001), agitation (OR = 2.331, 95% CI 1.041–5.218, *p* = 0.040), plasma exchange (OR = 4.654, 95% CI 1.042–20.782, *p* = 0.044), catheter dysfunction (OR = 10.528, 95% CI 2.939–37.710, *p* < 0.001) and out‐of‐unit transport for procedures (OR = 7.563, 95% CI 2.663–21.484, *p* < 0.001). The nomogram yielded AUCs of 0.789 (95% CI 0.726–0.841), 0.813 (95% CI 0.725–0.897) and 0.835 (95% CI 0.768–0.896) in the training, internal validation and external validation sets, respectively.

**Conclusions:**

Daily number of filter replacements, agitation, plasma exchange, catheter dysfunction and out‐of‐unit transport are independent risk factors for suboptimal CRRT downtime control. This nomogram showed satisfactory discrimination, calibration and net clinical benefit in internal and external validation, offering ICU nurses a rapid bedside risk assessment tool.

**Relevance to Clinical Practice:**

ICU nurses can use this nomogram at CRRT initiation to identify high‐risk patients and prioritise bedside procedures, sedation, catheter care and filter longevity to reduce downtime.

## Impact Statements

- What is known about the topic
  ○ CRRT downtime is a key quality indicator recommended by the 22nd Acute Disease Quality Initiative consensus, with a target of ≤ 2.4 h per 24 h.
  ○ Catheter dysfunction, filter clotting and anticoagulation strategies have been associated with prolonged CRRT downtime.
  ○ Existing studies have largely focused on unplanned CRRT interruption events and lack quantitative tools to assess whether daily cumulative downtime meets the recommended threshold.
- What this paper adds
  ○ This study developed a quantitative prediction model incorporating out‐of‐unit transport for procedures and plasma exchange, which were identified as independent factors associated with suboptimal CRRT downtime control.
  ○ Five bedside‐accessible indicators were integrated into a nomogram prediction model, enabling rapid bedside risk scoring.
  ○ A generalised estimating equation‐based approach was applied to account for within‐patient correlations across repeated CRRT treatment days.
  ○ Internal and external validation demonstrated satisfactory discrimination, calibration and clinical utility of the model.

## 1 Introduction

Continuous renal replacement therapy (CRRT) is an extracorporeal blood purification technique designed to operate continuously for 24 h or nearly 24 h per day, providing slow, continuous solute clearance and blood volume regulation. It offers distinct advantages for managing critically ill patients with haemodynamic instability [1, 2]. In the intensive care unit (ICU), the incidence of acute kidney injury (AKI) reaches 50%–60%, of whom 10%–15% require CRRT [3, 4].

Although CRRT is intended to be continuous, frequent treatment interruptions remain a pervasive problem [1]. CRRT downtime refers to the cumulative duration during which the blood pump ceases to rotate, caused by factors such as filter clotting, catheter dysfunction, out‐of‐unit transport for procedures and device alarms [5]. The 22nd Acute Disease Quality Initiative (ADQI) consensus explicitly identifies CRRT downtime as a key quality improvement indicator and recommends limiting downtime to ≤ 2.4 h per 24‐h period [6]. Rewa et al. [7] also included downtime among the 13 core quality indicators for CRRT through an international Delphi consensus process. However, clinical data indicate that downtime typically accounts for 22%–28% of treatment time [8], and a substantial gap remains between actual practice in most ICUs and the guideline recommendations.

Prolonged CRRT downtime leads to several adverse consequences. Effective solute clearance and electrolyte regulation are interrupted, potentially worsening acid–base imbalances [5, 8]. Treatment interruptions may also induce haemodynamic fluctuations, which are particularly hazardous [9, 10]. Frequent interruptions shorten filter lifespan, increase nursing workload and raise medical costs [11, 12]. Venkataraman et al. [13] showed that owing to frequent interruptions from filter clotting and out‐of‐unit transport, the actual delivered dose of CRRT was only 68% of the prescribed dose. Uchino et al. [1] reported a mean daily downtime of 3 h, which significantly and negatively correlated with the decrease in urea and creatinine. More importantly, Shin et al. [8] demonstrated that downtime ≥ 20% impaired fluid and uraemic control, exacerbated acidosis and interacted with daily fluid balance (*p* = 0.004), potentially increasing 28‐day mortality. Thus, reducing CRRT downtime is a core patient safety issue, and early identification of risk factors with targeted prevention is essential.

Recent studies have investigated factors influencing CRRT interruptions. The systematic review by Rewa et al. [14] identified 18 CRRT quality indicators, among which filter lifespan, small‐solute clearance and downtime were the three most frequently cited. Xia et al. [15] conducted a meta‐analysis of factors associated with CRRT interruption, including anticoagulation modality, vascular access type and filter clotting, but this analysis did not encompass non‐treatment‐related factors such as out‐of‐unit transport. Shin et al. [8] analysed the impact of downtime on clinical outcomes without establishing a quantifiable prediction model. Xu et al. [16] constructed a prediction model for unplanned CRRT interruption, but their focus was on whether an unplanned interruption occurred rather than on whether daily cumulative downtime met the recommended threshold. Currently, a quantitative risk assessment tool that can be embedded into the bedside workflow is lacking. Therefore, this study aimed to construct a visualised prediction model for suboptimal CRRT downtime control.

### 1.1 Aims of the Study

This study aimed to develop and validate a bedside‐accessible nomogram for early identification of treatment days at risk of suboptimal CRRT downtime control in critically ill patients. Specifically, we aimed to identify independent risk factors associated with suboptimal downtime control and develop a prediction model to support early risk assessment in clinical practice.

## 2 Methods

### 2.1 Study Design and Reporting Standards

This retrospective cohort study was conducted in the intensive care unit of a tertiary referral hospital in China, from January 2019 to December 2023. This time frame was selected because our centre has used a unified electronic medical record system to document CRRT treatment parameters since 2019. The study is reported in accordance with the STROBE and TRIPOD guidelines; the completed checklists are presented in Tables S1 and S2.

### 2.2 Participants

This study included adult critically ill patients admitted to the ICU between January 2019 and December 2023 who received CRRT. Inclusion criteria were as follows: (1) age ≥ 18 years; (2) received CRRT during ICU stay.

Exclusion criteria were as follows: (1) cumulative CRRT treatment time < 24 h, which does not allow a full 24‐h cycle for the outcome assessment; (2) incomplete medical records with > 20% missing data for any variable included in the final analysis (threshold chosen to ensure reliability of multivariable modelling); (3) treatment days with incomplete time boundaries, that is, a treatment day starting after 07:00 AM or ending before 07:00 AM the following day. The 07:00 cut‐off was chosen as the start and end point of a standardised treatment day because daily ICU nursing records at our centre close at 07:00 and laboratory tests are also drawn at 07:00. Aligning treatment day boundaries with this schedule ensures accuracy of downtime recording and consistency of daily laboratory collection times.

### 2.3 Sample Size Estimation

According to the sample size estimation principle for logistic regression analysis, 5–10 outcome events are required per predictor variable [17]. This study included 26 candidate variables. The minimum sample size for the suboptimal downtime group was approximately 145 treatment days, yielding a minimum total of 290 treatment days.

### 2.4 Data Partitioning

Data were partitioned chronologically into a model development cohort and an external validation cohort. The model development cohort comprised patients admitted between January 2019 and June 2022. Their treatment days were split into a training set and an internal validation set using stratified random sampling at a 7:3 ratio with the patient as the sampling unit, ensuring that all treatment days from a given patient belonged to the same dataset. This approach prevented data leakage across sets and avoided overestimation of internal validation performance. The external validation cohort consisted of patients admitted between July 2022 and December 2023, and was used to evaluate the temporal generalisability of the model.

### 2.5 Key Definitions

#### 2.5.1 CRRT Downtime

CRRT downtime was defined as the cumulative duration during which the blood pump ceased to rotate within a single standardised treatment day (07:00 to 07:00 the following day), irrespective of the cause. Causes included filter or tubing clotting necessitating filter replacement, catheter kinking, out‐of‐unit transport for procedures, device alarms, patient agitation, plasma exchange changeover and replacement fluid or dialysate bag changes. Permanent cessation due to death, withdrawal of treatment, transition to intermittent haemodialysis or attainment of treatment goals was not counted as downtime.

#### 2.5.2 Standardised Treatment Day

A standardised treatment day was defined as the 24 h period from 07:00 on a given day to 07:00 on the following day. Treatment days with non‐07:00 boundaries were excluded because they would produce incomplete‐24‐h cycles, introducing systematic bias. The 07:00 cut‐off was chosen because the daily ICU nursing records at the study hospital close at 07:00 and laboratory tests are also drawn at 07:00, ensuring accurate downtime recording and consistent laboratory collection times.

#### 2.5.3 Optimal Versus Suboptimal CRRT Downtime Control

A standardised treatment day with cumulative CRRT downtime ≤ 2.4 h was classified as optimal downtime control; > 2.4 h was classified as suboptimal control, based on the ADQI recommendation [6].

### 2.6 Variable Selection

From 41 candidate variables identified via literature review and expert discussion, 26 were retained after excluding unavailable or non‐variable items. Five time‐invariant baseline severity variables (age, sex, APACHE II, SOFA, BMI) were collected but excluded from selection because: (a) the unit of analysis was treatment day (these do not vary daily); (b) they reflect admission status, not modifiable daily factors; (c) the aim was to identify actionable daily risk factors.

The variables were grouped into four domains: (1) CRRT circuit and treatment parameters: catheter site, catheter dysfunction, treatment modality, daily number of filter replacements, filter type, anticoagulation; (2) patient clinical status on the treatment day: body temperature, agitation, daily duration of mechanical ventilation, daily duration of vasoactive drug infusion; (3) treatment management factors: out‐of‐unit transport for procedures, plasma exchange, daily ultrafiltration volume, daily fluid balance, daily blood product transfusion volume, haematocrit, haemoglobin, platelet count, D‐dimer, prothrombin time, activated partial thromboplastin time, fibrinogen, calcium concentration, serum creatinine, blood urea nitrogen; (4) nursing factor: mean seniority of responsible nurses.

### 2.7 Data Collection

Data were extracted from the hospital electronic medical record system (admission records, daily nursing notes, CRRT logs, laboratory reports and discharge summaries). Two trained ICU nurses independently extracted and cross‐checked data; disagreements were resolved by a third reviewer.

Baseline variables (age, sex, APACHE II, SOFA, BMI) came from admission records. Daily filter replacements were counted from CRRT logs. Key definitions: catheter dysfunction = pre‐pump pressure < −250 mmHg or inability to withdraw blood [18]; out‐of‐unit transport = leaving ICU for imaging/surgery during CRRT; plasma exchange = any plasma exchange on that day; agitation = RASS ≥ + 2 [19]. Laboratory values were obtained from the clinical lab system for the corresponding treatment day. Daily ultrafiltration, fluid balance and blood product volume were extracted from CRRT and intake‐output records. Mean nurse seniority was calculated from daily duty schedules.

### 2.8 Statistical Analysis

All statistical analyses were performed using Python 3.9. The distribution of continuous variables was assessed for normality using the Shapiro–Wilk test combined with visual inspection. Normally distributed continuous variables were described as mean ± standard deviation (SD), and between‐group comparisons were performed using the independent‐samples *t*‐test; non‐normally distributed continuous variables were described as median (P25, P75), and between‐group comparisons were performed using the Mann–Whitney *U* test. Categorical variables were presented as frequencies and percentages [*n* (%)], and between‐group comparisons were performed using the chi‐squared test, with Fisher's exact test used when expected frequencies were < 5. All statistical tests were two‐sided, and a *p* < 0.05 was considered statistically significant.

Generalised estimating equations (GEE) with an exchangeable correlation structure were used for univariable and multivariable logistic regression to account for within‐patient clustering. Variables with *p* < 0.05 in univariable analysis were entered into a multivariable GEE model, followed by backward stepwise elimination (removal criterion *p* ≥ 0.05) to identify independent risk factors for suboptimal CRRT downtime control. A nomogram was constructed from the final model's regression coefficients. Data partitioning followed Section 2.4. Model discrimination was assessed by the AUC (95% CI via bootstrap with 1000 resamples clustered by patient ID). Calibration was evaluated using calibration plots and the Brier score, and net clinical benefit by decision curve analysis.

To examine whether five baseline severity variables (age, sex, APACHE II, SOFA, BMI) affected model composition, a post hoc sensitivity analysis was performed in the full derivation cohort. And all five were forced in simultaneously. The stability of odds ratios for the primary risk factors was assessed by comparing the original model with each sensitivity model.

### 2.9 Ethics Statement

This study was approved by the Ethics Committee of the Fourth Hospital of Hebei Medical University (Approval No. 2023KS260, 26 December 2023). Informed consent was waived due to the retrospective nature of the study.

## 3 Results

### 3.1 Patient Characteristics

A total of 145 patients were included. The median age was 62.0 (51.0–71.0) years, 90 patients (62.1%) were male, the median APACHE II score was 21.0 (18.0–28.0), the median SOFA score was 10.0 (8.0–13.0) and the mean BMI was 23.08 ± 3.81 kg/m^2^ (Table 1). A flow diagram of patient and treatment‐day selection is provided in Figure S1.

**TABLE 1:** Baseline characteristics of the study population (*N* = 145).

Variables	Value (*n* = 145)
Age (years), median (P25, P75)	62.00 (51.00, 71.00)
Sex, *n* (%)	
Male	90 (62.1)
Female	55 (37.9)
BMI (kg/m^2^), mean ± SD	23.08 ± 3.81
APACHE II, median (P25, P75)	21.00 (18.00, 28.00)
SOFA, median (P25, P75)	10.00 (8.00, 13.00)

*Note:* Data are presented as mean ± standard deviation (SD), median (P25, P75), or *n* (%), as appropriate. BMI data were available for 97 patients due to missing measurements.

Abbreviations: APACHE II, acute physiology and chronic health evaluation II; BMI, body mass index; SOFA, sequential organ failure assessment.

### 3.2 Comparison of Baseline Characteristics Between the Model Development and External Validation Cohorts

The model development cohort (*n* = 112) and the external validation cohort (*n* = 33) showed no statistically significant differences in sex, age, APACHE II score, SOFA score or BMI (all *p* > 0.05) (Table 2), indicating homogeneous baseline severity between time periods.

**TABLE 2:** Comparison of baseline characteristics between the model development cohort and the external validation cohort.

Variables	Model development cohort (*n* = 112)	External validation cohort (*n* = 33)	Statistic	*p*
Sex (male), *n* (%)	65 (58.0)	25 (75.8)	2.689^a^	0.101
Age (years), median (P25, P75)	62.00 (51.00, 70.00)	64.00 (51.00, 73.00)	1759.500^b^	0.678
APACHE II score, median (P25, P75)	22.00 (17.75, 28.25)	21.00 (20.00, 27.00)	1745.000^b^	0.628
SOFA score, median (P25, P75)	11.00 (8.00, 14.00)	10.00 (8.00, 11.00)	2100.000^b^	0.234
BMI (kg/m^2^), mean ± SD	23.34 ± 3.68	22.45 ± 4.10	1.046^c^	0.298

*Note:* BMI data were partially missing: complete data were available for 69 patients in the model development cohort and 28 patients in the external validation cohort. Within the model development cohort, 78 patients were assigned to the training set and 34 to the internal validation set; all 33 patients in the external validation cohort were independent patients.

^a^ Chi‐squared test.

^b^ Mann–Whitney *U* test.

^c^ Independent samples *t*‐test.

### 3.3 Current Status of CRRT Downtime

A total of 595 standardised treatment days from 145 patients were analysed, yielding a cumulative treatment time of 14 280 h and a cumulative downtime of 1725.07 h. The mean downtime per treatment day was (2.90 ± 2.72 h), accounting for (12.08% ± 11.35%) of total daily treatment time. Using 2.4 h as the threshold, 232 treatment days (39.0%) had suboptimal CRRT downtime control.

### 3.4 Univariable GEE Analysis

With suboptimal CRRT downtime control as the dependent variable, univariable GEE analysis was performed in the training set (Table S3). Eight variables showed statistically significant differences (*p* < 0.05), seven of which were associated with increased risk of suboptimal CRRT downtime control (OR > 1): catheter dysfunction (OR = 11.963, 95% CI 3.154–45.383, *p* < 0.001) conferred the highest risk, followed by out‐of‐unit transport for procedures (OR = 9.796, 95% CI 3.818–25.135, *p* < 0.001), plasma exchange (OR = 6.318, 95% CI 1.382–28.895, *p* = 0.017), daily number of filter replacements (OR = 3.224, 95% CI 2.299–4.521, *p* < 0.001), agitation (OR = 2.314, 95% CI 1.134–4.722, *p* = 0.021), platelet count (OR = 1.004, 95% CI 1.001–1.006, *p* = 0.005) and daily ultrafiltration volume (OR = 1.000, 95% CI 1.000–1.000, *p* = 0.046). Calcium concentration (OR = 0.297, 95% CI 0.096–0.923, *p* = 0.036) was the only variable showing a negative association, which is inconsistent with previous studies; this was further evaluated in multivariable analysis.

### 3.5 Multivariable GEE Analysis

After backward stepwise selection, five independent risk factors were retained (Table 3). Catheter dysfunction had the strongest effect (OR = 10.528, 95% CI 2.939–37.710, *p* < 0.001), followed by out‐of‐unit transport for procedures (OR = 7.563, 95% CI 2.663–21.484, *p* < 0.001), plasma exchange (OR = 4.654, 95% CI 1.042–20.782, *p* = 0.044), daily number of filter replacements (OR = 2.629, 95% CI 1.822–3.794, *p* < 0.001) and agitation (OR = 2.331, 95% CI 1.041–5.218, *p* = 0.040). The remaining three variables lost significance after adjustment, suggesting that their significance in the univariable analysis may have been influenced by confounding.

**TABLE 3:** Multivariable GEE regression analysis of independent risk factors for suboptimal CRRT downtime control.

Variables	β	SE	*Z* value	*p*	OR	95% CI lower	95% CI upper
Daily number of filter replacements	0.967	0.187	5.17	< 0.001	2.629	1.822	3.794
Agitation (yes vs. no)	0.846	0.411	2.06	0.040	2.331	1.041	5.218
Plasma exchange (yes vs. no)	1.538	0.763	2.01	0.044	4.654	1.042	20.782
Catheter dysfunction (yes vs. no)	2.354	0.651	3.62	< 0.001	10.528	2.939	37.710
Out‐of‐unit transport for procedures (yes vs. no)	2.023	0.533	3.80	< 0.001	7.563	2.663	21.484

*Note:* GEE with exchangeable correlation structure and logit link function.

### 3.6 Sensitivity Analysis

Adding the five baseline severity variables individually or simultaneously to the final model did not materially alter the odds ratios of the primary risk factors (all changes < 6%), and none of the severity variables were statistically significant (Tables S4 and S5).

### 3.7 Nomogram Construction

**FIGURE 1:**
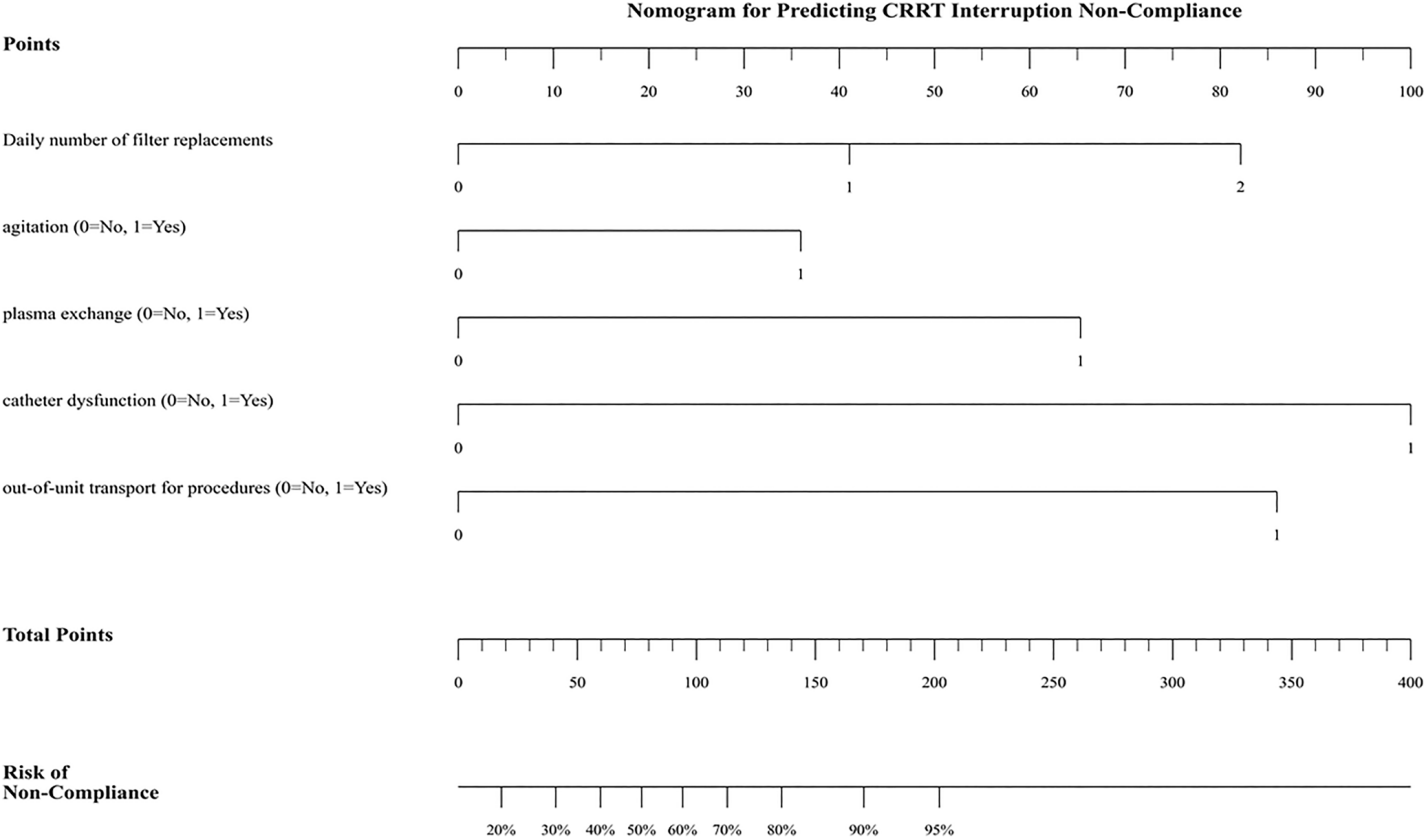
Nomogram for predicting suboptimal CRRT downtime control.

A nomogram was constructed based on the five independent risk factors (Figure 1).

### 3.8 Model Performance

**FIGURE 2:**
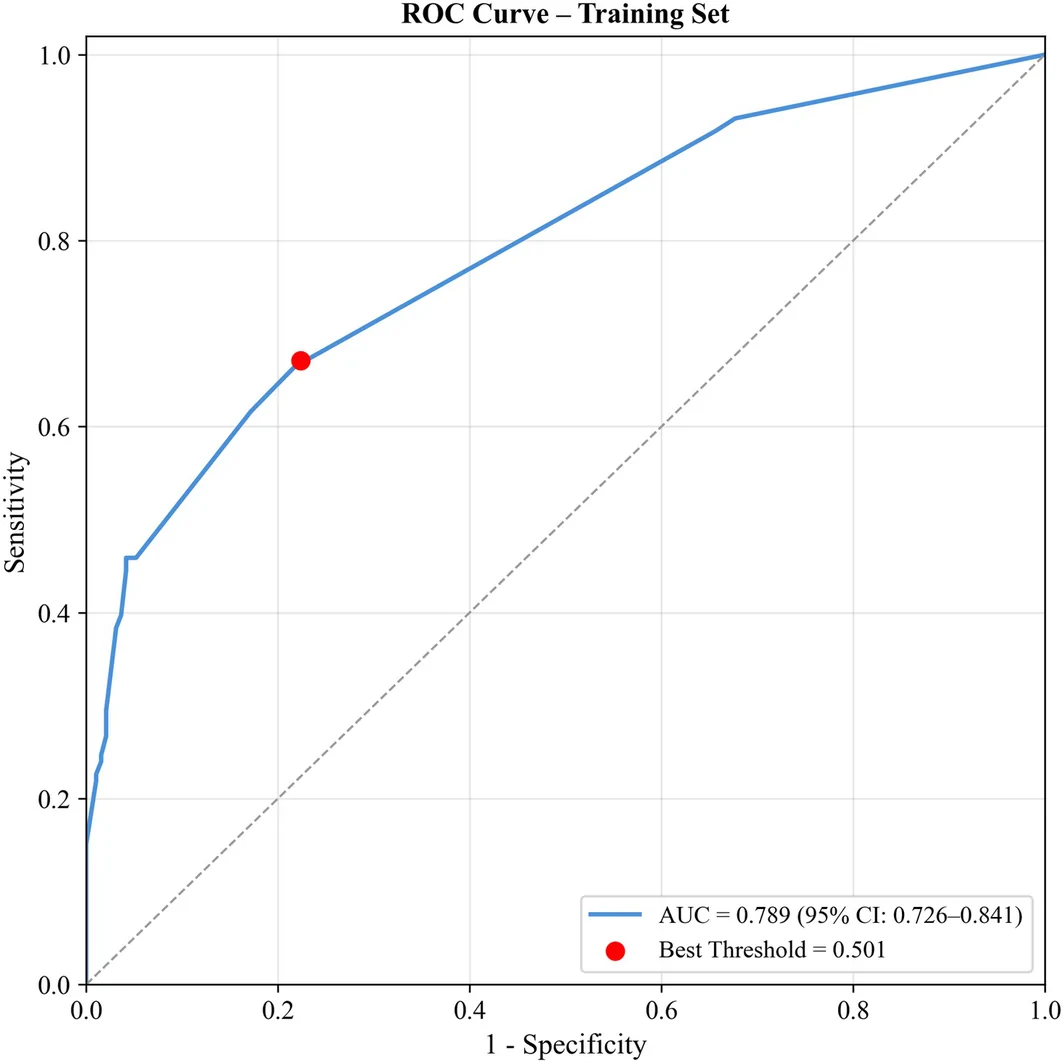
AUC of the training set.

**FIGURE 3:**
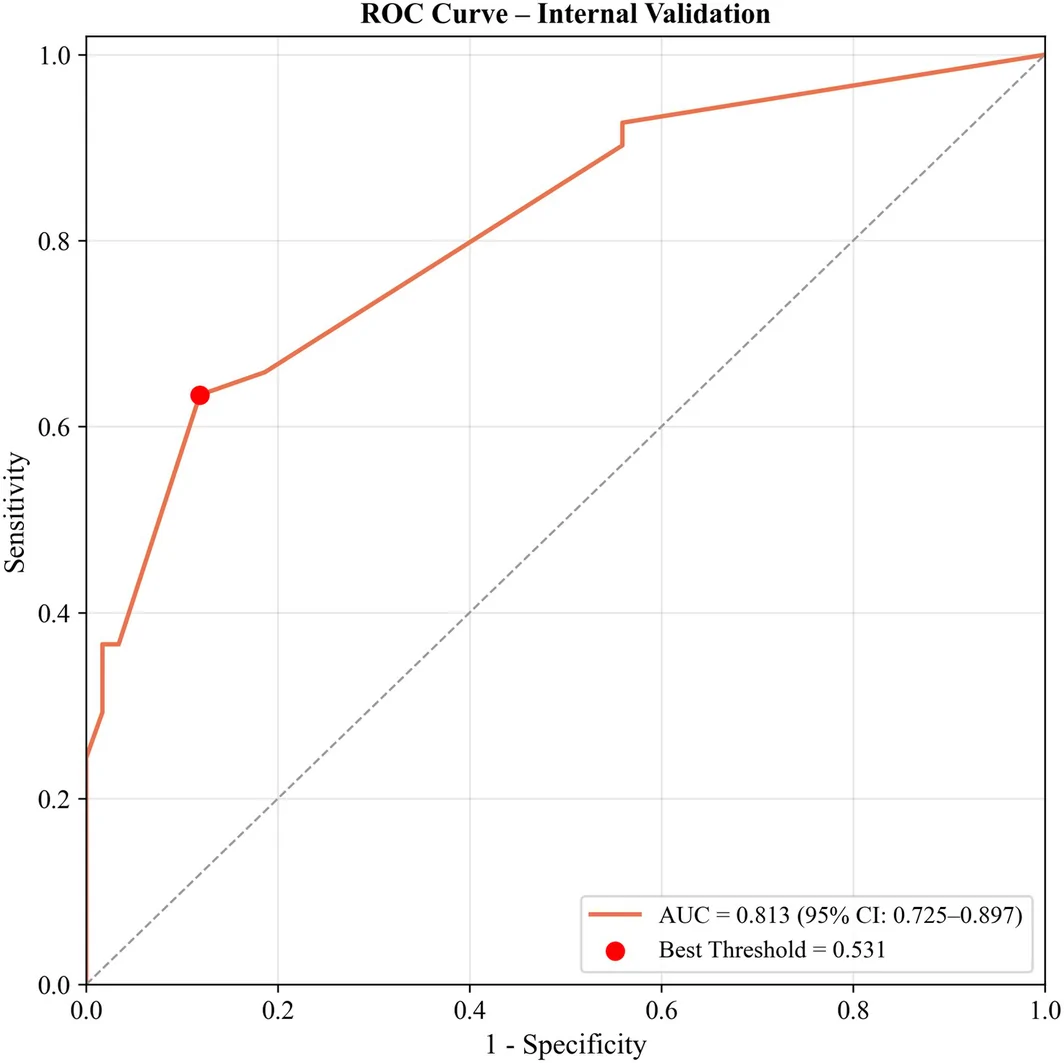
AUC of the internal validation set.

**FIGURE 4:**
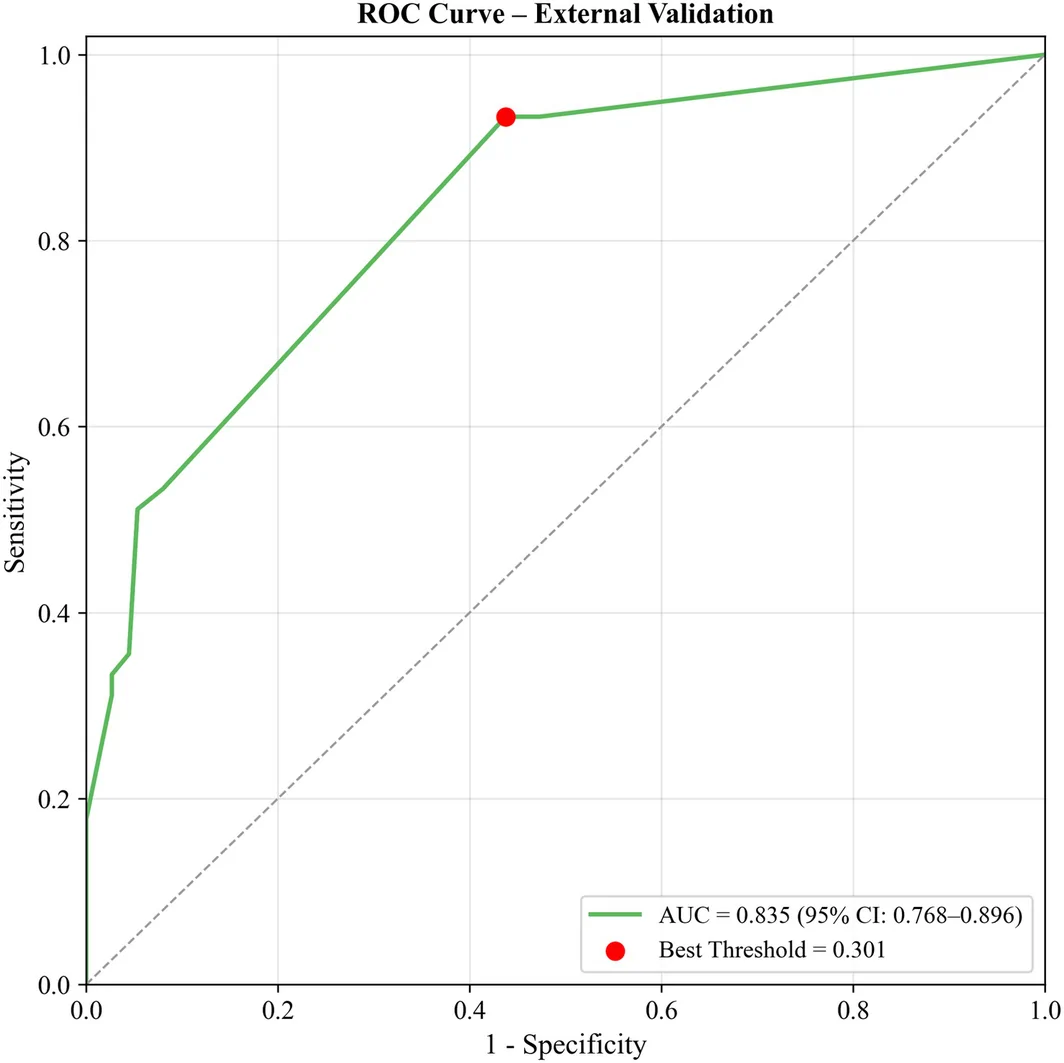
AUC of the external validation set.

**FIGURE 5:**
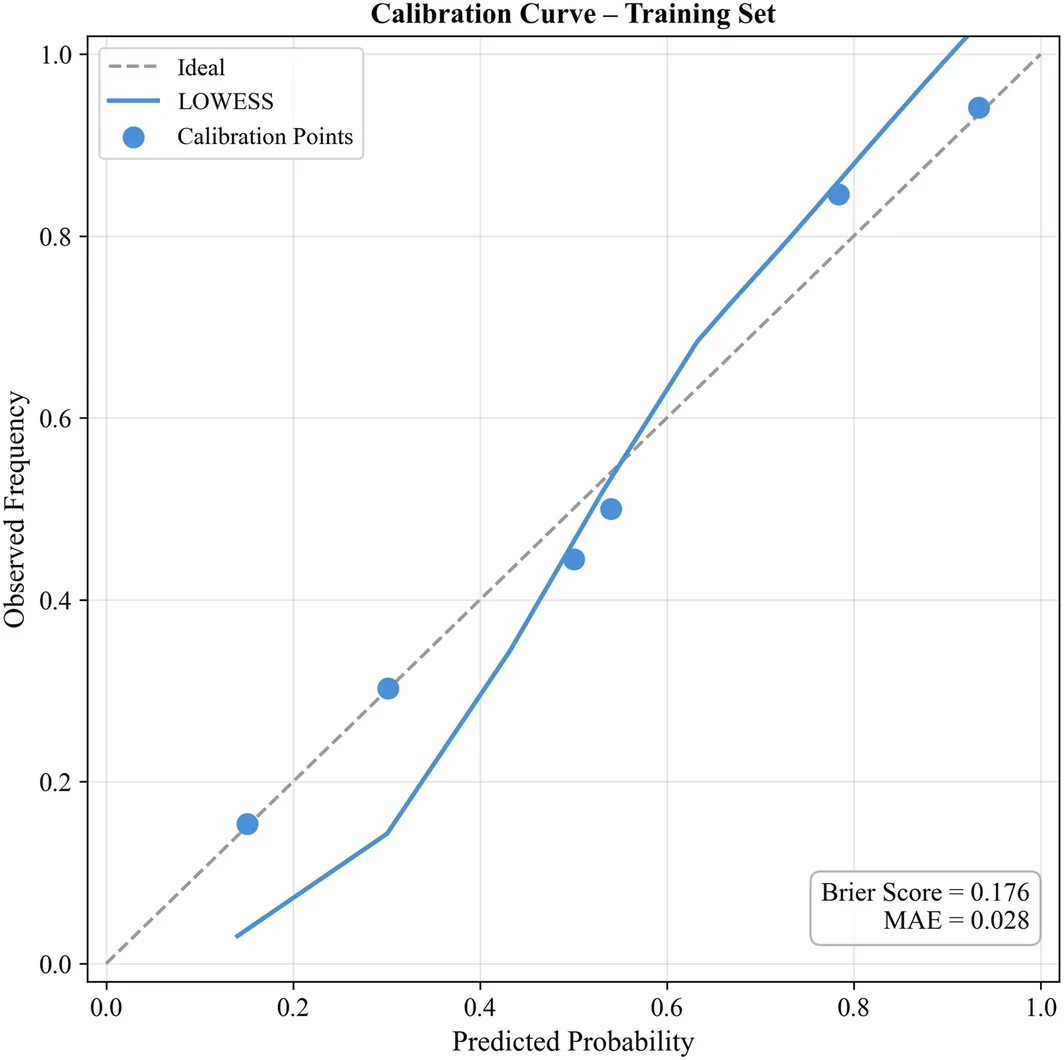
Calibration curve of the training set.

**FIGURE 6:**
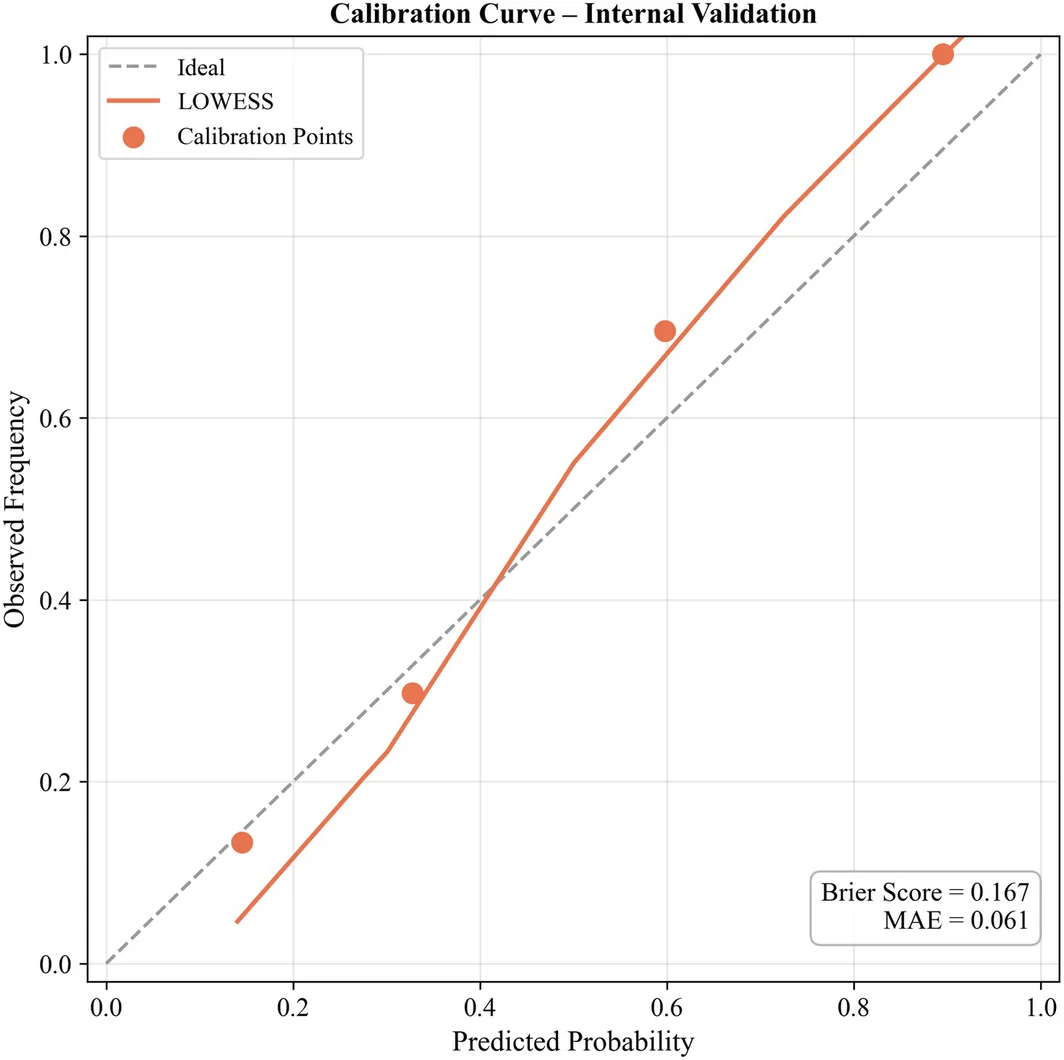
Calibration curve of the internal validation set.

**FIGURE 7:**
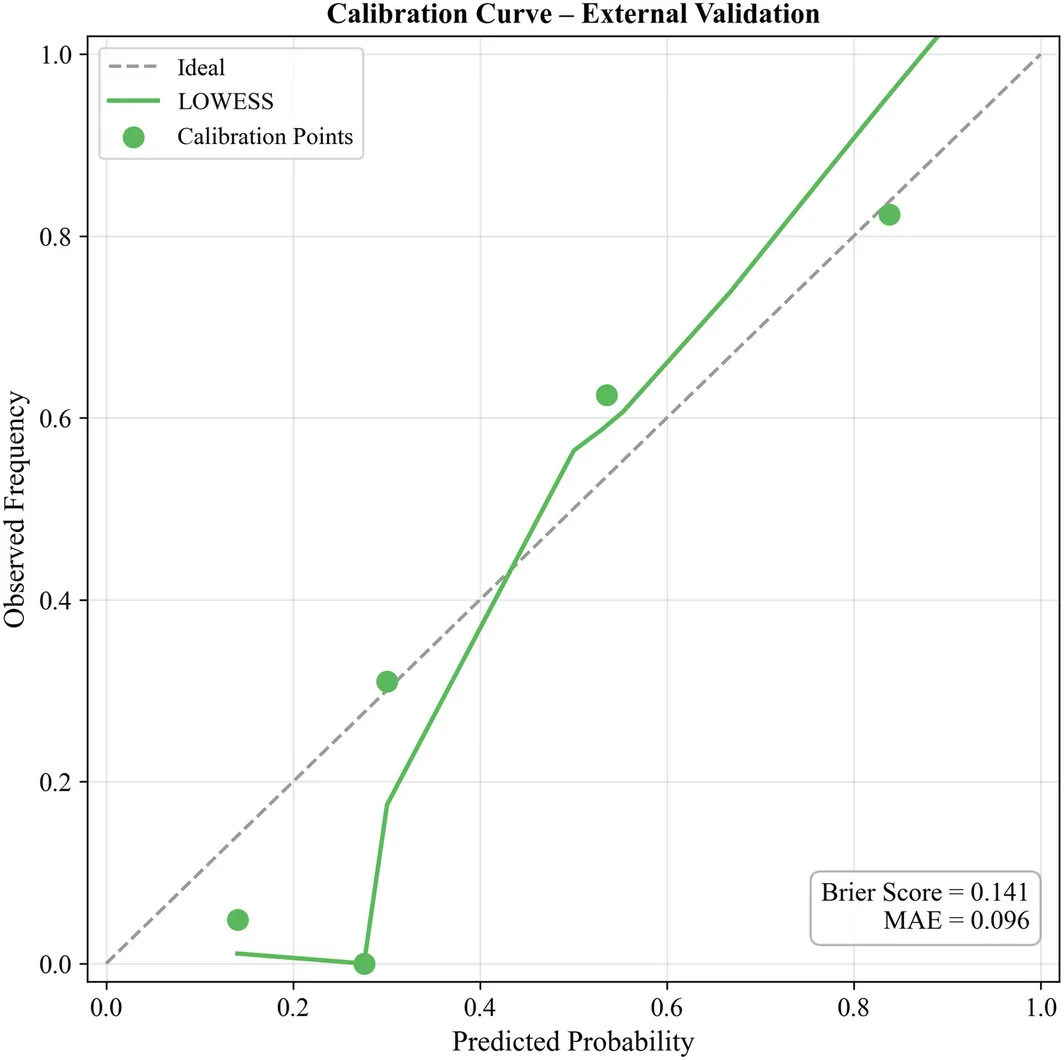
Calibration curve of the external validation set.

**FIGURE 8:**
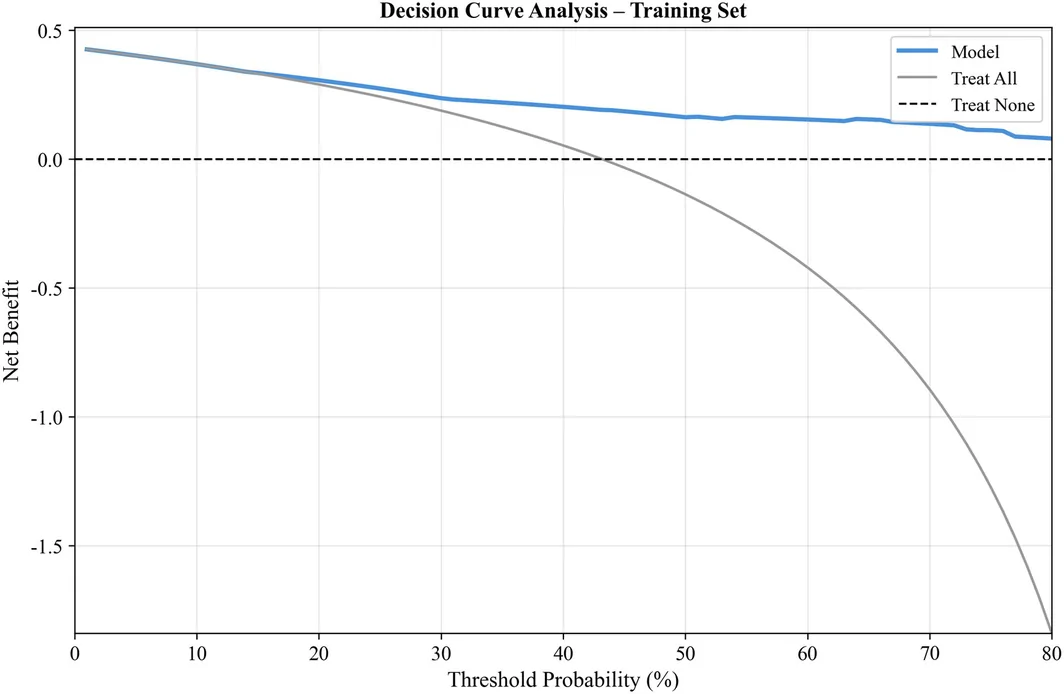
Decision curve of the training set.

**FIGURE 9:**
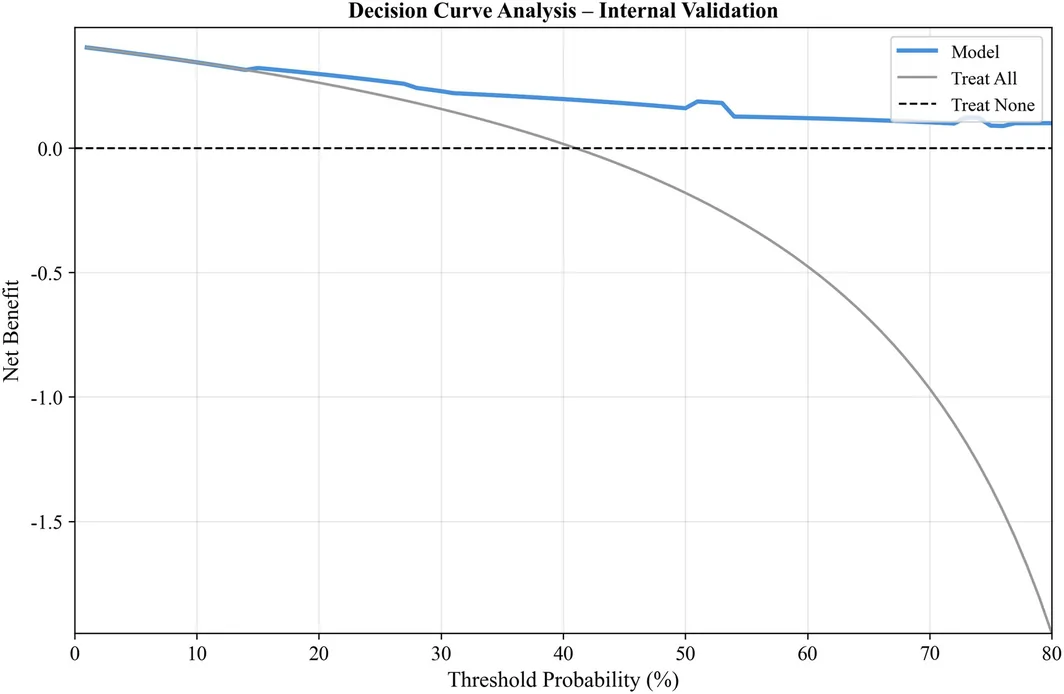
Decision curve of the internal validation set.

**FIGURE 10:**
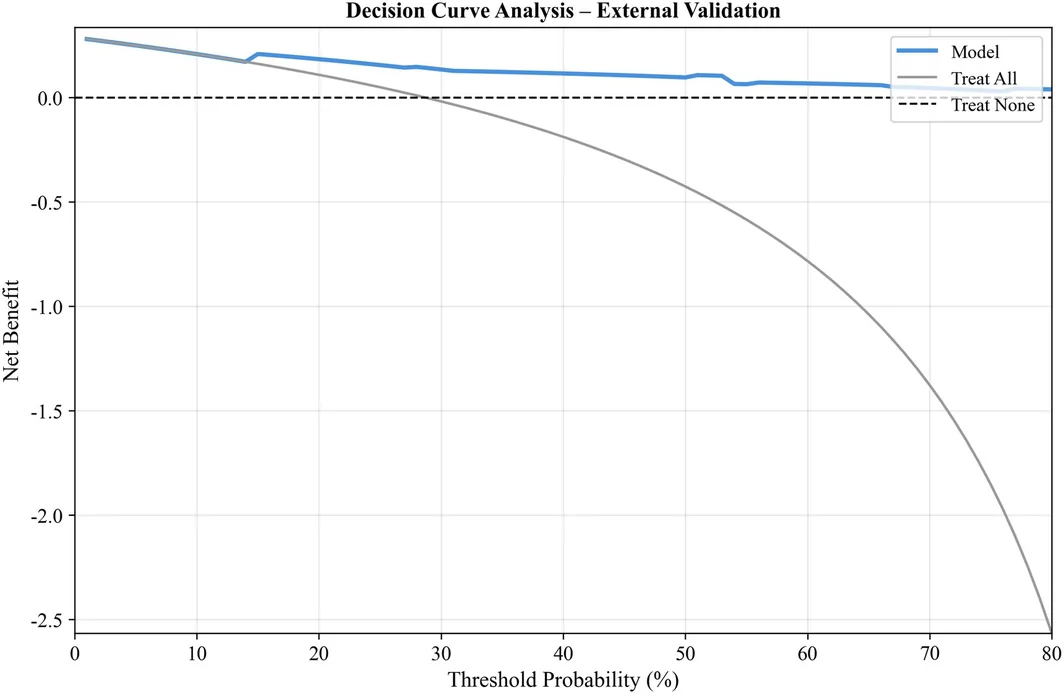
Decision curve of the external validation set.

The AUC was 0.789 (95% CI 0.726–0.841) in the training set, 0.813 (95% CI 0.725–0.897) in the internal validation set and 0.835 (95% CI 0.768–0.896) in the external validation set (Figures 2, 3, 4). Calibration curves showed good agreement between predicted and observed probabilities, with Brier scores of 0.195 (training), 0.168 (internal) and 0.159 (external) (Figures 5, 6, 7). Decision curve analysis demonstrated net benefit over threshold probabilities of 10%–50% in all three sets, with the external validation set showing the greatest net benefit (Figures 8, 9, 10).

## 4 Discussion

### 4.1 Summary of Key Findings

In 595 treatment days from 145 critically ill patients, the mean daily CRRT downtime was 2.90 h, and 39.0% of treatment days failed to meet the ADQI‐recommended standard of ≤ 2.4 h. Catheter dysfunction, out‐of‐unit transport for procedures, plasma exchange, daily number of filter replacements and agitation were identified as independent risk factors for suboptimal CRRT downtime control. The nomogram constructed from these five indicators yielded an AUC of 0.835 in external validation, demonstrating satisfactory discrimination and clinical utility.

### 4.2 Current Status of CRRT Downtime

The mean proportion of daily downtime (12.08%) was substantially lower than the 25.6% reported by Shin et al. in Korea [8], which may reflect our centre's standardised circuit priming and anticoagulation monitoring protocols. Nevertheless, 39.0% of treatment days still exceeded the recommended threshold. Prolonged downtime compromises fluid management and metabolic control. Braun et al. in the United States [20] found that in patients with high alarm frequency (≥ 6 alarms/day), treatment downtime was positively associated with the fluid balance gap (β = 0.87, *p* < 0.001). Shin et al. [8] further showed that downtime ≥ 20% impaired fluid and uraemic control, with an interaction between downtime and daily fluid balance (*p* = 0.004). These findings indicate that reducing downtime is critical for maintaining treatment quality.

### 4.3 Mechanisms Underlying the Risk Factors

Catheter dysfunction was the strongest independent risk factor in this study (OR = 10.528), consistent with reports by Xia et al. in China [15] and Kim et al. in Australia [21]. It interrupts treatment through two main mechanisms. First, when the catheter tip abuts the vessel wall or kinks, withdrawal pressure drops sharply (<‐250 mmHg), triggering protective pump cessation. Second, repeated obstruction creates turbulence and blood stasis that accelerate filter clotting. Kim et al. [21] further reported that approximately 13.6% of filter shutdowns were attributable to mechanical catheter dysfunction, with patient repositioning and agitation‐induced displacement as major predisposing factors. The 2012 KDIGO guideline [22] recommends the right internal jugular vein as the preferred temporary access site, followed by the femoral and left internal jugular veins.

Out‐of‐unit transport for procedures (OR = 7.563) is another important finding of this study, and it has rarely been examined as an independent variable in previous research on unplanned CRRT interruption. The mechanism is straightforward: patients must be disconnected from the CRRT device and transported to the radiology department or operating theatre. The entire process‐transport preparation, disconnection during transit, waiting and reconnection‐results in cumulative downtime that far exceeds that from other causes. Zhao et al. in China [5] reported a median single‐transport downtime of 1.64 h (IQR 1.20–3.26 h), substantially longer than the bag‐change time of 1.17 ± 0.66 h, and noted that downtime caused by out‐of‐unit transport had the largest within‐group variability. Venkataraman et al. in the United States [13] also confirmed that transport‐related downtime was one of the main reasons for CRRT interruption.

Plasma exchange was identified as a novel risk factor in this study (OR = 4.654) and has received limited attention in previous research. The mechanism is straightforward: the CRRT circuit typically must be paused during plasma exchange, directly causing unavoidable downtime. Foglia et al. in the United States [23] demonstrated in a paediatric ICU that a tandem CRRT and therapeutic plasma exchange strategy reduced the median proportion of CRRT downtime from 14.7% (IQR 10.5%–26%) to 3.4% (IQR 1.3%–4.9%) (*p* < 0.001).

Daily number of filter replacements (OR = 2.629) indirectly reflects the cumulative effect of filter lifespan on downtime. Each filter replacement entails blood return and disconnection, circuit installation, priming and re‐initiation, with a mean total time of approximately 58 min as reported from China [24]; more frequent replacements therefore lead to greater cumulative downtime. Filter clotting, the main reason for replacement, is associated with anticoagulation strategy, blood flow rate and transmembrane pressure management. Yue et al. in China [25] compared three anticoagulation strategies in burn patients and found the median filter lifespan was 31.06 h with citrate, significantly longer than 14.08 h with heparin and 16.42 h with nafamostat. Zhou et al. in China [26] further confirmed in a network meta‐analysis that regional citrate anticoagulation extends filter lifespan (MD 12.0 h) and reduces bleeding risk.

Agitation (OR = 2.331) has been confirmed as an independent risk factor for unplanned CRRT interruption in multiple studies. The mechanisms may involve two aspects: first, agitation leads to frequent changes in body position, causing kinking, abutting or even accidental dislodgement of the vascular access catheter, directly triggering blood pump alarm cessation; second, the sympathetic excitation and muscle activity associated with agitation may increase blood viscosity and coagulation tendency, indirectly accelerating filter clotting. A meta‐analysis by Zeng et al. in China [27], including 23 studies, 3793 patients and 7197 filters, confirmed that agitation was an independent risk factor for unplanned CRRT weaning (OR = 4.97, 95% CI 3.20–7.74).

### 4.4 Innovations and Strengths of This Study

This study has three main innovations. First, from a research perspective, it moves beyond the previous focus on unplanned interruption events by using the ADQI‐recommended 2.4‐h threshold as the outcome and treating downtime as a quantifiable nursing quality indicator, which aligns more closely with the needs of clinical quality management. Second, with respect to predictor expansion, this study incorporated out‐of‐unit transport for procedures and plasma exchange into a quantitative prediction model for suboptimal CRRT downtime control, thereby expanding knowledge of non‐treatment‐related factors. Third, methodologically, GEE was used to account for correlations among multiple treatment days within the same patient, avoiding the parameter estimation bias that may result from ignoring the data clustering structure inherent in conventional logistic regression. Temporal external validation was also performed, providing a more realistic assessment of the model's generalisability across different time periods. The systematic review by Zhang et al. in China [28] indicated that existing prediction models primarily focus on circuit lifespan, with AUCs ranging from 0.679 to 0.997; our model, which targets a quality indicator threshold as the prediction endpoint, represents an advance in this field. Post hoc sensitivity analysis confirmed that adding the five baseline severity variables individually or simultaneously did not alter the model's composition (all *p* > 0.05, OR changes < 6%), further supporting the robustness of a modelling strategy focused on modifiable daily risk factors.

### 4.5 Relevance to Clinical Practice

The nomogram developed in this study integrates five bedside‐accessible indicators into an intuitive scoring tool. For patients with a high predicted probability, nurses can implement targeted preventive measures at CRRT initiation: (1) communicate with the medical team to prioritise bedside ultrasound or mobile CT as alternatives to out‐of‐unit transport, and if transport is unavoidable, plan the route in advance to minimise downtime; (2) comprehensively assess vascular access function, including blood withdrawal patency and return pressure, and perform ultrasound‐guided catheter position assessment when necessary; (3) on the basis of the prescribed anticoagulation regimen, increase the frequency of anticoagulation monitoring and closely observe changes in filter and circuit pressures and colour to extend filter lifespan; (4) regularly assess sedation depth using the RASS, collaborate with physicians to optimise the sedation regimen, reinforce catheter and tubing securement, and prevent agitation‐related interruptions; (5) for treatment days on which plasma exchange is planned, prepare the necessary supplies in advance and actively adopt a tandem treatment mode to compress the changeover window.

These findings are consistent with the CRRT quality dashboard concept proposed by Mottes et al. in the United States [29] and the structured quality assurance system described by Ruiz et al. in the United States [30], both of which improve outcomes through continuous monitoring of process indicators. Embedding this nomogram into electronic health information systems could, in combination with artificial intelligence technologies, enable automated alerts and intelligent clinical decision support, as envisioned by Cheungpasitporn et al. in the United States [31]. Such tools, however, should be positioned to assist rather than replace clinical judgement, and their successful application requires human oversight and rigorous multicentre validation.

### 4.6 Limitations

This study has several limitations. First, it is a single‐centre retrospective study; although external validation yielded favourable results, the model's generalisability and transportability still require multicentre prospective verification. Second, owing to limitations of the electronic medical record system, real‐time circuit pressure parameters such as transmembrane pressure and filter pressure could not be included. Third, although GEE accounted for correlations among multiple treatment days from the same patient, estimation efficiency may decline when the number of treatment days per patient varies substantially, even though GEE is generally robust to unbalanced data. Fourth, this study focused on the process indicator of suboptimal CRRT downtime control and did not examine the direct association between downtime and long‐term outcomes such as ICU mortality, 28‐day mortality or renal recovery, which limits the ability to link the model to patient endpoint events. Fifth, the 95% CI for plasma exchange was wide (1.042–20.782), likely reflecting the limited number of exposed cases; the stability of this effect therefore requires verification in larger samples. Sixth, total calcium rather than ionised calcium was used, and BMI data were missing in 32.9% of cases, which may have limited the completeness of baseline characteristic descriptions.

### 4.7 Future Research Directions

Future research could proceed in several directions. First, multicentre prospective cohort studies across diverse regions and hospital types should externally validate and, if necessary, recalibrate the model. Second, real‐time machine data such as transmembrane pressure trends and blood pump speed variability should be incorporated to enable dynamic risk assessment. Third, longer follow‐up should directly link downtime with hard endpoints, including 28‐day mortality and renal recovery rate, to clarify the clinical benefit of reducing downtime. Finally, AI technologies could be integrated into the model to support automated early warning and intelligent clinical decision‐making.

## 5 Conclusion

In this cohort of CRRT patients, the mean daily downtime was 2.90 h, and 39.0% of treatment days failed to meet the ADQI‐recommended standard of 2.4 h. A greater daily number of filter replacements, agitation, undergoing plasma exchange, catheter dysfunction and out‐of‐unit transport for procedures are independent risk factors for suboptimal CRRT downtime control. The nomogram prediction model constructed based on these factors and generalised estimating equations demonstrated satisfactory discrimination, calibration and net clinical benefit in both internal and external validation. This model can provide critical care nurses with an evidence‐based tool for early identification of high‐risk treatment days and formulation of individualised prevention strategies at the bedside.

## Funding

The study was funded by the Health Commission of Hebei Province Project (No. 20210797).

## Ethics Statement

This study was approved by the Ethics Committee of the Fourth Hospital of Hebei Medical University (Approval No. 2023KS260, 26 December 2023) prior to its commencement.

## Consent

As this study was retrospective in nature, written informed consent was waived.

## Conflicts of Interest

The authors declare no conflicts of interest.

## Supporting information


**Figure S1:** Flow chart of patient screening and grouping.

**Table S1:** STROBE Statement—Checklist of items that should be included in reports of cohort studies.
**Table S2:** TRIPOD checklist: prediction model development and validation.
**Table S3:** Univariable GEE analysis of factors associated with suboptimal CRRT downtime control in the training set.
**Table S4:** Sensitivity analysis: baseline severity variables added to the final model.
**Table S5:** Comparison of odds ratios for the five risk factors between the original model and the full model (including all five baseline variables).

## Data Availability

The data that support the findings of this study are available from the corresponding author upon reasonable request.
