# Supplementary material for: Analysis of Factors Influencing Downtime During Continuous Renal Replacement Therapy in Critically Ill Patients and Construction of a Prediction Model
Source: Nurs Crit Care. 2026 Aug 27;31(5):e70664. doi: 10.1111/nicc.70664 (PMC13522733; doi:10.1111/nicc.70664)
Supplement: Supplementary file 1 [file NICC-31-0-s001.docx]

Note: Downtime threshold of 2.4 hours is based on ADQI recommendation.

**Figure S1. Flowchart of patient screening and grouping**
