# Supplementary material for: Analysis of Factors Influencing Downtime During Continuous Renal Replacement Therapy in Critically Ill Patients and Construction of a Prediction Model
Source: Nurs Crit Care. 2026 Aug 27;31(5):e70664. doi: 10.1111/nicc.70664 (PMC13522733; doi:10.1111/nicc.70664)
Supplement: Supplementary file 2 [file NICC-31-0-s002.doc]

**Table S1. STROBE Statement — Checklist of items that should be included in reports of cohort studies**

| Item No | Recommendation | Reported on Page/Section |
| --- | --- | --- |
| **Title and abstract** | | |
| 1 | (a) Indicate the study's design with a commonly used term in the title or the abstract | Abstract: "This retrospective cohort study developed and validated a prediction model" |
|  | (b) Provide in the abstract an informative and balanced summary of what was done and what was found | Abstract: Background, Aims, Study Design, Methods, Results, Conclusions, Relevance to Clinical Practice |
| **Introduction** | | |
| 2 | Background/rationale: Explain the scientific background and rationale for the investigation being reported | Introduction ¶1–4: CRRT downtime as a pervasive clinical problem; ADQI consensus recommending ≤2.4 h per 24 h; gap between guideline and clinical practice; adverse consequences of prolonged downtime including impaired solute clearance, haemodynamic fluctuations, and increased mortality risk |
| 3 | Objectives: State specific objectives, including any prespecified hypotheses | Section 1.1 (Aims): Four specific objectives: (1) describe current distribution of CRRT downtime; (2) screen for independent risk factors for suboptimal downtime control; (3) construct and validate a prediction model; (4) visualize the model as a nomogram |
| **Methods** | | |
| 4 | Study design: Present key elements of study design early in the paper | Section 2.1: Retrospective cohort study; January 2019 to December 2023; intensive care unit of a tertiary referral hospital in China; reported in accordance with STROBE statement |
| 5 | Setting: Describe the setting, locations, and relevant dates, including periods of recruitment, exposure, follow-up, and data collection | Section 2.1: Study period January 2019–December 2023; rationale for timeframe (unified electronic medical record system since 2019). Section 2.5.2: Standardised treatment day defined as 07:00 to 07:00 the following day. Section 2.4: Data partitioned chronologically into model development cohort (January 2019–June 2022) and external validation cohort (July 2022–December 2023) |
| 6 | (a) Cohort study—Give the eligibility criteria, and the sources and methods of selection of participants. Describe methods of follow-up | Section 2.2: Inclusion criteria: (1) admitted to ICU between January 2019 and December 2023; (2) received CRRT; (3) aged ≥18 years. Exclusion criteria: (1) cumulative CRRT treatment time <24 h; (2) incomplete medical records with >20% missing data for key variables; (3) incomplete time boundaries of the standardised treatment day. Source: hospital electronic medical record system. Retrospective design, no prospective follow-up |
|  | (b) For matched studies, give matching criteria and number of exposed and unexposed | Not applicable (not a matched study) |
| 7 | Variables: Clearly define all outcomes, exposures, predictors, potential confounders, and effect modifiers. Give diagnostic criteria, if applicable | Section 2.5: Outcome: cumulative downtime >2.4 h per standardised treatment day (≤2.4 h defined as optimal, >2.4 h as suboptimal, per ADQI consensus). Section 2.6–2.7: 26 candidate predictors across four domains; key definitions provided (catheter dysfunction: pre-pump arterial pressure < −250 mmHg; agitation: RASS ≥+2; out-of-unit transport; plasma exchange) |
| 8 | Data sources/measurement: For each variable of interest, give sources of data and details of methods of assessment (measurement). Describe comparability of assessment methods if there is more than one group | Section 2.6: All data extracted from hospital electronic medical record system (admission records, daily nursing records, CRRT treatment records, laboratory test reports, discharge summaries). Two trained ICU nurses independently extracted data; cross-checked; disagreements adjudicated by a third reviewer. Specific extraction methods described for each variable category |
| 9 | Bias: Describe any efforts to address potential sources of bias | Section 2.8: GEE with exchangeable correlation structure to account for clustering effect of repeated measures within patients. Section 2.4: Data partitioning with patient as the sampling unit to prevent data leakage across sets |
| 10 | Study size: Explain how the study size was arrived at | Section 2.3: Sample size estimation based on 5–10 outcome events per predictor variable. 26 candidate variables required approximately 145 suboptimal treatment days; equal number for optimal group; minimum total 290 treatment days. Final analysis included 595 treatment days (232 suboptimal), exceeding minimum requirement |
| 11 | Quantitative variables: Explain how quantitative variables were handled in the analyses. If applicable, describe which groupings were chosen and why | Section 2.8: Continuous variables assessed for normality using Shapiro–Wilk test; normally distributed described as mean ± SD (t-test); non-normally distributed as median (P25, P75) (Mann–Whitney U test). Categorical variables as frequencies and percentages (χ² or Fisher's exact test). 2.4 h threshold based on ADQI consensus (≤10% of 24 h period) |
| 12 | (a) Describe all statistical methods, including those used to control for confounding | Section 2.8: Univariable and multivariable GEE logistic regression (exchangeable correlation structure, logit link); backward stepwise elimination (retention P<0.05). Model discrimination: AUC (95% CI via bootstrap with clustering by patient ID, 1000 resamples). Calibration: calibration curves, Brier score. Net clinical benefit: decision curve analysis |
|  | (b) Describe any methods used to examine subgroups and interactions | Not applicable |
|  | (c) Explain how missing data were addressed | Section 2.8: Patients with >20% missing data for key variables were excluded. For the primary analysis, all analyses were based on original data without imputation except for BMI, where missing values (32.9%) were handled by median imputation. For the post-hoc sensitivity analysis (Section 2.8 and Supplementary Table S4), median imputation for BMI was also applied. |
|  | (d) Cohort study—If applicable, explain how loss to follow-up was addressed | Not applicable (retrospective design, daily cross-sectional unit of observation) |
|  | (e) Describe any sensitivity analyses | Section 2.8 (Methods) & Section 3.6 (Results) & Supplementary Table S4. A post‑hoc sensitivity analysis was performed to assess whether omission of five baseline severity variables (age, sex, APACHE II, SOFA, BMI) affected model composition. Each variable was added individually and simultaneously to the final multivariable GEE model using the full derivation cohort after median imputation. None were significant (all P > 0.05), and the odds ratios of the five identified risk factors remained stable (all changes <6%). |
| **Results** | | |
| 13 | (a) Report numbers of individuals at each stage of study—e.g., numbers potentially eligible, examined for eligibility, confirmed eligible, included in the study, completing follow-up, and analysed | Section 3.1–3.3 and Supplementary Figure S1: 231 patients assessed → 86 excluded (3 missing data, 2 age <18, 81 CRRT <24 h) → 145 patients eligible → 287 treatment days excluded (144 start after 07:00, 143 end before 07:00 next day) → 595 treatment days included in final analysis |
|  | (b) Give reasons for non-participation at each stage | Section 2.2 and Supplementary Figure S1: Detailed exclusion reasons provided |
|  | (c) Consider use of a flow diagram | Supplementary Figure S1: Flow diagram of patient and treatment-day selection provided |
| 14 | (a) Give characteristics of study participants (e.g., demographic, clinical, social) and information on exposures and potential confounders | Section 3.1 (Table 1): Mean age 59.73 ± 15.63 years, 62.1% male, APACHE II 22.92 ± 8.56, SOFA 10.63 ± 4.04, BMI 23.08 ± 3.81 kg/m². Section 3.2 (Table 2): Comparison of model development and external validation cohorts |
|  | (b) Indicate number of participants with missing data for each variable of interest | Table 1 note: BMI missing for 48/145 patients (33.1%). All other variables had complete data |
| 15 | Report numbers of outcome events or summary measures over time | Section 3.3: 595 treatment days, 14,280 h total treatment time, 1,725.07 h cumulative downtime; mean daily downtime 2.90 ± 2.72 h (12.08% ± 11.35% of daily treatment time); 232 treatment days (39.0%) with suboptimal downtime control; stratified: 138 (23.2%) with 2.4–4.8 h, 43 (7.2%) with 4.8–7.2 h, 51 (8.6%) with >7.2 h |
| 16 | (a) Give unadjusted estimates and, if applicable, confounder-adjusted estimates and their precision (e.g., 95% confidence interval). Make clear which confounders were adjusted for and why they were included | Section 3.4 (Table S3): Univariable GEE analysis for all 26 candidate variables (OR, 95% CI, P values). Section 3.5 (Table 3): Multivariable GEE analysis: five independent risk factors retained (daily number of filter replacements OR=2.629; agitation OR=2.331; plasma exchange OR=4.654; catheter dysfunction OR=10.528; out-of-unit transport OR=7.563; all P<0.05). Backward stepwise elimination used for variable selection |
|  | (b) Report category boundaries when continuous variables were categorized | Section 2.5.3: 2.4 h threshold based on ADQI consensus. Other continuous variables kept as continuous in analysis |
|  | (c) If relevant, consider translating estimates of relative risk into absolute risk for a meaningful time period | Not applicable |
| 17 | Other analyses—Report other analyses done (e.g., analyses of subgroups and interactions, and sensitivity analyses) | Section 3.7: Model performance assessed by AUC (training: 0.789; internal validation: 0.813; external validation: 0.835), calibration curves, Brier scores, and decision curve analysis. Section 3.6 and Supplementary Table S4: Sensitivity analysis for baseline severity variables (age, sex, APACHE II, SOFA, BMI). |
| **Discussion** | | |
| 18 | Summarise key results with reference to study objectives | Section 4.1: 39.0% of treatment days had suboptimal downtime control; catheter dysfunction strongest risk factor (OR=10.528); out-of-unit transport (OR=7.563) and plasma exchange (OR=4.654) newly identified; external validation AUC=0.835 |
| 19 | Discuss limitations of the study, taking into account sources of potential bias or imprecision. Discuss both direction and magnitude of any potential bias | Section 4.6: Six limitations: (1) single-centre retrospective design; (2) unavailability of real-time circuit pressure parameters; (3) GEE estimation efficiency concern with varying treatment day numbers; (4) focus on process indicator without direct association with long-term outcomes; (5) wide 95% CI for plasma exchange due to limited exposed cases; (6) total calcium rather than ionised calcium used, and BMI missing in 32.9% of cases handled by median imputation. |
| 20 | Give a cautious overall interpretation of results considering objectives, limitations, multiplicity of analyses, results from similar studies, and other relevant evidence | Section 4.2–4.5: Interpretation situated within existing literature; downtime proportion (12.08%) lower than Shin et al. (median 25.6%); catheter dysfunction mechanism discussed with reference to Kim et al. (2010) and KDIGO guidelines; novel risk factors discussed with Zhao et al. (2025) and Foglia et al. (2021) |
| 21 | Discuss the generalisability (external validity) of the study results | Section 4.6: Single-centre design limits generalisability; external validation (33 independent patients from different time period) yielded AUC 0.835, supporting temporal transportability; multicentre prospective studies needed |
| **Other information** | | |
| 22 | Funding: Give the source of funding and the role of the funders for the present study and, if applicable, for the original study on which the present article is based | Title page:Funding section: "This study was supported by the Health Commission of Hebei Province Project (No. 20210797). The funder had no role in study design, data collection, data analysis, manuscript preparation, or the decision to submit for publication." |

### **Table S 2. TRIPOD Checklist: Prediction Model Development and Validation**

| Section/Topic | Item No | Checklist Item (D=Development; V=Validation) | Reported on Page/Paragraph |
| --- | --- | --- | --- |
| **Title and Abstract** | | | |
| Title | 1 | Identify the study as developing and/or validating a multivariable prediction model, the target population, and the outcome to be predicted. (D;V) | **Title:** "Analysis of factors influencing downtime during continuous renal replacement therapy in critically ill patients and construction of a prediction model"  Target population: critically ill patients.  Outcome: downtime during continuous renal replacement therapy. |
| Abstract | 2 | Provide a summary of objectives, study design, setting, participants, sample size, predictors, outcome, statistical analysis, results, and conclusions. (D;V) | **Abstract:** All required elements are reported, including Background, Aims, Study Design, Results (AUC, calibration), and Conclusions. |
| **Introduction** | | | |
| Background and Objectives | 3a | Explain the medical context (including whether diagnostic or prognostic) and rationale for developing or validating the multivariable prediction model, including references to existing models. (D;V) | **Introduction ¶1-4:** Medical context is CRRT adequacy and quality. Rationale: Existing studies lack a quantitative bedside tool to predict whether daily downtime meets the ADQI-recommended ≤2.4 h threshold. References to existing work (Uchino 2003, Shin 2022, Xu 2024) are provided. |
| Background and Objectives | 3b | Specify the objectives, including whether the study describes the development or validation of the model or both. (D;V) | **Section 1.1 (Aims):** Objectives 2 & 3 specifically state "screen for independent risk factors... construct a prediction model and validate it using internal and external data." |
| **Methods** | | | |
| Source of Data | 4a | Describe the study design or source of data (e.g., randomized trial, cohort, or registry data), separately for the development and validation data sets, if applicable. (D;V) | **Section 2.1 & 2.4:** A retrospective cohort study. Data source is a single-centre ICU. The model development cohort (Jan 2019-Jun 2022) and external validation cohort (Jul 2022-Dec 2023) are described separately. |
| Source of Data | 4b | Specify the key study dates, including start of accrual; end of accrual; and, if applicable, end of follow-up. (D;V) | **Section 2.1 & 2.4:** Study period: January 2019 to December 2023. Development cohort: Jan 2019-Jun 2022. External validation cohort: Jul 2022-Dec 2023. No follow-up as it's a daily cross-sectional analysis. |
| Participants | 5a | Specify key elements of the study setting (e.g., primary care, secondary care, general population) including number and location of centres. (D;V) | **Section 2.1:** "...conducted in the intensive care unit of a tertiary referral hospital in China." Single centre. |
| Participants | 5b | Describe participant eligibility criteria. (D;V) | **Section 2.2:** Inclusion: admitted to ICU, received CRRT, ≥18 years. Exclusion: CRRT <24h, >20% missing data, incomplete treatment day boundaries. |
| Participants | 5c | Give details of treatments received, if relevant. (D;V) | **Introduction ¶1 & Methods:** All participants received continuous renal replacement therapy (CRRT). |
| Outcome | 6a | Clearly define the outcome that is predicted by the prediction model, including how and when assessed. (D;V) | **Section 2.5.1 & 2.5.3:** Outcome is suboptimal CRRT downtime control, defined as cumulative blood pump cessation >2.4 h per standardised treatment day (07:00 to 07:00). |
| Outcome | 6b | Report any actions to blind assessment of the outcome to be predicted. (D;V) | Not applicable. The outcome is an objective, machine-recorded measurement (blood pump cessation time) extracted from electronic records. |
| Predictors | 7a | Clearly define all predictors used in developing or validating the multivariable prediction model, including how and when they were measured. (D;V) | **Section 2.6 & 2.7:** All 26 candidate predictors are defined (e.g., agitation: RASS ≥+2; catheter dysfunction: pre-pump arterial pressure < -250 mmHg). Measurement timing is specified (e.g., lab values from the corresponding treatment day). |
| Predictors | 7b | Report any actions to blind assessment of predictors for the outcome and other predictors. (D;V) | Not applicable. Predictors are objective clinical/lab data or documented events extracted retrospectively by independent reviewers, blinded to the study hypothesis. |
| Sample Size | 8 | Explain how the study size was arrived at. (D;V) | **Section 2.3:** Sample size estimated using rule of 5-10 events per variable. 26 candidates required ~145 events; final analysis includes 232 suboptimal days. |
| Missing Data | 9 | Describe how missing data were handled with details of any imputation. (D;V) | **Section 2.8:** "All analyses were based on original data without imputation of missing values." Patients with >20% missing key data were excluded. |
| Statistical Analysis Methods | 10a | Describe how predictors were handled in the analyses. (D) | **Section 2.8:** Continuous predictors were kept continuous; categorical predictors were coded as dummy variables. GEE was used to handle within-patient correlations. |
| Statistical Analysis Methods | 10b | Specify type of model, all model-building procedures , and method for internal validation. (D) | **Section 2.8:** GEE logistic regression model. Backward stepwise selection. Internal validation via 7:3 stratified random split with patient as the sampling unit. |
| Statistical Analysis Methods | 10c | For validation, describe how the predictions were calculated. (V) | **Section 2.8 & 3.7:** The nomogram (Fig 1) and final GEE model coefficients were applied to the validation datasets to calculate predicted probabilities. |
| Statistical Analysis Methods | 10d | Specify all measures used to assess model performance and, if relevant, to compare multiple models. (D;V) | **Section 2.8 & 3.7:** Discrimination (AUC, 95% CI via bootstrap), calibration (calibration curve, Brier score), and net clinical benefit (decision curve analysis). |
| Statistical Analysis Methods | 10e | If done, describe any model updating (e.g., recalibration) arising from the validation. (V) | Not done. |
| Risk Groups | 11 | Provide details on how risk groups were created, if done. (D;V) | Not done. |
| Development vs. Validation | 12 | For validation, identify any differences in the setting, eligibility criteria, outcome, and predictors between development and validation datasets. (V) | **Section 3.2 (Table 2):** No significant differences in baseline characteristics (age, sex, APACHE II, SOFA, BMI) between development and external validation cohorts. |
| **Results** | | | |
| Participants | 13a | Describe the flow of participants through the study, including the number of participants with and without the outcome and. A flow diagram is strongly recommended. (D;V) | **Section 3.1-3.3:** 145 patients → 595 treatment days → 232 (39.0%) suboptimal days. Patient flow is described; A flow diagram of patient and treatment-day selection is provided in Supplementary Figure S1. |
| Participants | 13b | Describe the characteristics of the participants (basic demographics, clinical features, available predictors), including the number of participants with missing data for predictors and outcome. (D;V) | **Section 3.1 (Table 1) & Section 3.2 (Table 2):** Demographics and clinical features provided. Missing BMI data for 48/145 patients is reported in Table 1. |
| Participants | 13c | For validation, show a comparison of the distribution of important variables (demographics, predictors, and outcome) between development and validation datasets. (V) | **Section 3.2 (Table 2):** Direct comparison of age, sex, APACHE II, SOFA, and BMI between the two cohorts. |
| Model Development | 14a | Specify the number of participants and outcome events in each analysis. (D) | **Section 3.4 (Table S3):** 146 suboptimal and 192 optimal. |
| Model Development | 14b | If done, report the unadjusted association between each candidate predictor and outcome. (D) | **Section 3.4 (Table S3):** Univariable GEE analysis (OR, 95% CI, P-value) reported for all 26 candidate variables. |
| Model Specification | 15a | Present the full prediction model to allow predictions for individuals (i.e., all regression coefficients, and model intercept or baseline survival at a given time point). (D) | **Section 3.5 (Table 3):** Final model coefficients (β) are reported. The nomogram (Figure 1) serves as the visual full model for predictions. |
| Model Specification | 15b | Explain how to the use the prediction model. (D) | **Section 3.6:** Instructions for using the nomogram are provided ("By drawing a vertical line upward... the predicted probability..."). |
| Model Performance | 16 | Report performance measures (with confidence intervals) for the prediction model. (D;V) | **Section 3.7:** AUC: Training 0.789 (0.726–0.841), Internal validation 0.813 (0.725–0.897), External validation 0.835 (0.768–0.896). Calibration curves and Brier scores reported. |
| Model Updating | 17 | If done, report the results from any model updating (i.e., model specification, model performance). (V) | Not applicable. |
| **Discussion** | | | |
| Limitations | 18 | Discuss any limitations of the study . (D;V) | **Section 4.6:** Six limitations discussed: (1) single-centre retrospective design; (2) unavailability of real-time circuit pressure parameters; (3) GEE estimation efficiency concern; (4) focus on process indicator without direct association with long-term outcomes; (5) wide 95% CI for plasma exchange; (6) total calcium rather than ionised calcium, and BMI missing data handled by median imputation. |
| Interpretation | 19a | For validation, discuss the results with reference to performance in the development data, and any other validation data. (V) | **Section 4.1 & 4.4:** External validation AUC (0.835) is compared favorably with the training set (0.789), supporting temporal generalizability. |
| Interpretation | 19b | Give an overall interpretation of the results, considering objectives, limitations, results from similar studies, and other relevant evidence. (D;V) | **Section 4.1-4.5:** Summary of key findings, mechanism discussion (catheter dysfunction, transport), and comparison with previous literature (e.g., Shin 2022, Zhao 2025) are provided. |
| Implications | 20 | Discuss the potential clinical use of the model and implications for future research. (D;V) | **Section 4.5 & 4.7:** Clinical use for bedside risk assessment and targeted prevention (e.g., coordinating bedside procedures) is described. Future research directions including multicentre validation and AI integration are proposed. |
| **Other Information** | | | |
| Supplementary Information | 21 | Provide information about the availability of supplementary resources, such as study protocol, web calculator, and data sets. (D;V) | **STROBE Checklist note:** A flow diagram and STROBE checklist are provided in the supplementary material. |
| Funding | 22 | Give the source of funding and the role of the funders for the present study. (D;V) | **Funding section:** "This study was supported by the Health Commission of Hebei Province Project (No. 20210797). The funder had no role in study design, data collection... or the decision to submit for publication." |

**Table S3 Univariable GEE analysis of factors associated with suboptimal CRRT downtime control in the training set**

| Variable | Suboptimal downtime group (training set, n = 146) | Optimal downtime group (training set, n = 192) | Statistic | OR | 95%CI lower | 95%CI upper | P value (GEE) |
| --- | --- | --- | --- | --- | --- | --- | --- |
| haematocrit | 0.28 (0.25, 0.31) | 0.28 (0.25, 0.30) | 13079.000 (U) | 21.194 | 0.424 | 1059.865 | 0.126 |
| haemoglobin | 91.15 (85.03, 102.78) | 92.25 (85.25, 99.83) | 13319.500 (U) | 1.007 | 0.994 | 1.021 | 0.267 |
| Platelet count | 119.00 (58.50, 165.75) | 71.00 (37.00, 150.50) | 11066.500 (U) | 1.004 | 1.001 | 1.006 | 0.005 |
| D-dimer | 3.63 (2.13, 7.11) | 3.89 (1.51, 7.81) | 13773.500 (U) | 0.991 | 0.980 | 1.003 | 0.125 |
| PT | 12.80 (11.72, 14.20) | 13.20 (12.20, 15.65) | 16204.500 (U) | 0.960 | 0.897 | 1.027 | 0.239 |
| APTT | 33.90 (29.30, 40.65) | 34.05 (29.80, 40.73) | 14381.000 (U) | 1.005 | 0.998 | 1.012 | 0.140 |
| Fibrinogen | 4.10 (2.98, 4.94) | 3.54 (2.50, 4.80) | 12303.500 (U) | 1.148 | 0.978 | 1.348 | 0.092 |
| Calcium concentration | 2.08 (1.98, 2.18) | 2.09 (2.00, 2.21) | 15623.000 (U) | 0.297 | 0.096 | 0.923 | 0.036 |
| Creatinine | 150.85 (106.32, 211.12) | 129.30 (84.70, 187.03) | 12296.500 (U) | 1.000 | 0.999 | 1.002 | 0.605 |
| Blood urea nitrogen | 7.55 (5.60, 10.60) | 7.40 (5.50, 9.80) | 13042.500 (U) | 1.015 | 0.982 | 1.050 | 0.373 |
| Body temperature | 36.88 ± 0.59 | 36.72 ± 0.66 | -2.169 (t) | 1.361 | 0.958 | 1.932 | 0.085 |
| Daily duration of mechanical ventilation (h) | 24.00 (0.00, 24.00) | 24.00 (0.00, 24.00) | 14379.500 (U) | 0.997 | 0.977 | 1.017 | 0.748 |
| Daily duration of vasoactive agent use (h) | 10.50 (0.00, 24.00) | 21.00 (0.00, 24.00) | 14859.000 (U) | 0.994 | 0.972 | 1.016 | 0.609 |
| Daily volume of blood products (ml) | 100.00 (0.00, 327.50) | 280.00 (0.00, 420.00) | 15707.500 (U) | 1.000 | 0.999 | 1.001 | 0.470 |
| Daily fluid removal | 2099.00 (1313.00, 2717.00) | 2251.00 (1534.75, 2818.75) | 14805.000 (U) | 1.000 | 1.000 | 1.000 | 0.046 |
| Daily fluid balance | -410.00 (-1010.00, 117.00) | -399.00 (-993.00, 159.00) | 14540.000 (U) | 1.000 | 1.000 | 1.000 | 0.794 |
| Number of filter changes per day | 1.00 (1.00, 2.00) | 1.00 (0.00, 1.00) | 7792.000 (U) | 3.224 | 2.299 | 4.521 | <0.001 |
| Mean years of experience of primary nurse | 8.00(5.75,9.67) | 8.33 (6.33, 9.67) | 12999.000 (U) | 0.981 | 0.894 | 1.077 | 0.685 |
| Agitation (yes) | 29 (20.0%) | 16 (8.3%) | 8.736 (χ²) | 2.314 | 1.134 | 4.722 | 0.021 |
| Plasma exchange performed (yes) | 9 (6.2%) | 2 (1.0%) | Fisher | 6.318 | 1.382 | 28.895 | 0.017 |
| Catheter dysfunction (yes) | 19 (13.0%) | 2 (1.0%) | 18.397 (χ²) | 11.963 | 3.154 | 45.383 | <0.001 |
| Transport out of ICU for examination (yes) | 35 (24.0%) | 5 (2.6%) | 34.275 (χ²) | 9.796 | 3.818 | 25.135 | <0.001 |
| Filter type | 123 (84.2%) | 170 (88.5%) | 0.980 (χ²) | 0.646 | 0.301 | 1.389 | 0.263 |
| Anticoagulation use (yes) | 96 (65.8%) | 98 (51.0%) | 6.751 (χ²) | 1.463 | 0.927 | 2.309 | 0.102 |
| Mode (reference = CVVHD) | — | — | — | — | — | — | — |
| CVVH | 77 (52.7%) | 83 (43.2%) | 3.374 (χ²) | 2.269 | 0.842 | 6.113 | 0.105 |
| CVVHDF | 65 (44.5%) | 100 (52.1%) | — | 1.706 | 0.628 | 4.634 | 0.294 |
| Catheter site (reference = right internal jugular) | — | — | — | — | — | — | — |
| Right femoral vein | 92 (63.0%) | 131 (68.2%) | 2.546 (χ²) | 0.723 | 0.388 | 1.349 | 0.308 |
| Left femoral vein | 23 (15.8%) | 33 (17.2%) | — | 0.795 | 0.330 | 1.912 | 0.608 |
| Left internal jugular | 0 (0.0%) | 0 (0.0%) | — | — | — | — | — |

Note: Continuous variables are presented as median (P25, P75), except body temperature (mean ± SD). Categorical variables are presented as n (%). The letter in parentheses after the statistic indicates the test method: U (Mann-Whitney U test), t (t-test), χ² (Chi-square test), Fisher (Fisher's exact test). Univariable GEE analysis was performed in the training set (n = 338 treatment days; 146 suboptimal and 192 optimal).

**Table S4. Sensitivity analysis: baseline severity variables added to the final model**

| Variable | OR (95% CI) | P value |
| --- | --- | --- |
| Age (per year) | 1.006 (0.985–1.028) | 0.569 |
| Sex (male vs. female) | 0.843 (0.502–1.416) | 0.519 |
| APACHE II (per point) | 1.008 (0.979–1.038) | 0.608 |
| SOFA (per point) | 1.006 (0.951–1.064) | 0.840 |
| BMI (per kg/m²) | 1.004 (0.944–1.067) | 0.906 |

Note: Results from separate GEE models adding each variable to the five risk factors (filter replacements, agitation, plasma exchange, catheter dysfunction, out‑of‑unit transport). All P > 0.05. Missing values for BMI (32.9%) were handled by median imputation.

**Supplementary Table S5. Comparison of odds ratios for the five risk factors between the original model and the full model (including all five baseline variables)**

| Risk factor | OR in original model | OR in full model | Absolute change |
| --- | --- | --- | --- |
| Daily number of filter replacements | 2.902 | 2.948 | +0.046 (+1.6%) |
| Agitation | 1.957 | 2.018 | +0.061 (+3.1%) |
| Plasma exchange | 5.000 | 5.297 | +0.297 (+5.9%) |
| Catheter dysfunction | 11.217 | 11.429 | +0.212 (+1.9%) |
| Out‑of‑unit transport | 8.198 | 8.408 | +0.210 (+2.6%) |

Note: The full model includes all five baseline severity variables (age, sex, APACHE II, SOFA, BMI) simultaneously. All changes in OR were <6%, indicating stability of the risk factor estimates. Missing values for BMI (32.9%) were handled by median imputation.
